# A Dual Functional Artificial SEI Layer Based on a Facile Surface Chemistry for Stable Lithium Metal Anode

**DOI:** 10.3390/molecules27165199

**Published:** 2022-08-15

**Authors:** Yue Ma, Feng Wu, Nan Chen, Tianyu Yang, Yaohui Liang, Zhaoyang Sun, Guangqiu Luo, Jianguo Du, Yanxin Shang, Mai Feng, Ziyue Wen, Li Li, Renjie Chen

**Affiliations:** 1 Beijing Key Laboratory of Environmental Science and Engineering, School of Materials Science and Engineering, Beijing Institute of Technology, Beijing 100081, China; 2 Advanced Technology Research Institute, Beijing Institute of Technology, Jinan 250300, China; 3 Collaborative Innovation Center of Electric Vehicles in Beijing, Beijing 100081, China; 4 The 18th Research Institute of China Electronics Technology Group Corporation, Tianjin 300384, China

**Keywords:** lithium metal anode, dual functional, artificial SEI layer, Li-Mg alloy, lithium fluoride, lithium dendrite

## Abstract

Solid electrolyte interphase (SEI) on a Li anode is critical to the interface stability and cycle life of Li metal batteries. On the one hand, components of SEI with the passivation effect can effectively hinder the interfacial side reactions to promote long-term cycling stability. On the other hand, SEI species that exhibit the active site effect can reduce the Li nucleation barrier and guide Li deposition homogeneously. However, strategies that only focus on a separated effect make it difficult to realize an ideal overall performance of a Li anode. Herein, a dual functional artificial SEI layer simultaneously combining the passivation effect and the active site effect is proposed and constructed via a facial surface chemistry method. Simultaneously, the formed LiF component effectively passivates the anode/electrolyte interface and contributes to the long-term stable cycling performance, while the Li-Mg solid solution alloy with the active site effect promotes the transmission of Li^+^ and guides homogeneous Li deposition with a low energy barrier. Benefiting from these advantages, the Li||Li cell with the modified anode performs with a lower nucleation overpotential of 2.3 mV, and an ultralong cycling lifetime of over 2000 h at the current density of 1 mA cm^−2^, while the Li||LiFePO_4_ full battery maintains a capacity retention of 84.6% at rate of 1 C after 300 cycles.

## 1. Introduction

Lithium metal batteries (LMBs) are extensively studied as promising high-energy-density storage device because of their high theoretical capacity, low density, and also the low electrochemical potential of Li metal [1,2,3]. However, the practical application of rechargeable LMBs is limited by the formation of Li dendrites due to the inhomogeneous plating of Li^+^ during cycling. The “dead Li” detaches from the dendrites, accelerating capacity decay and increasing the internal resistance of the battery. Moreover, excessive growth of lithium dendrites cuts through the separator, causing short circuits and safety issues. The abovementioned issues result in low Coulombic efficiency (CE), shortened cycle life, and can cause fires or even battery explosion. This becomes a severe safety hazard for LMBs [4,5].

To date, various kinds of approaches have been reported to suppress harmful dendritic prolongation on Li anodes, such as the structural design of anode materials [6,7], the introduction of artificial protective layers [8,9], enhancement of the solid electrolyte interphase (SEI) on the Li metal anode [10,11], utilization of a gel polymer/solid-state electrolyte [12,13], as well as modification of the separator [14,15]. As the electrochemical potential of metallic Li is lower than that of the electrolyte, Li metal reacts spontaneously with the organic components in the electrolyte, forming unstable and fragile SEI generated on the Li anode. The properties of SEI strongly affect Li plating/stripping behavior, which is essential for the practical application possibilities of LMBs.

Many strategies have been researched to introduce artificial SEI formed ex situ or in situ on the Li anode to improve its stability. One important strategy is to alleviate its high activity using electrolyte additives (lithium nitrate, fluoroethylene carbonate, and aluminum oxide [16,17,18,19]) to generate chemically stable interface in situ to avoid side reactions, or preparing an artificial SEI layer with fluorine or nitrogen-containing components (lithium fluoride in poly (vinylidene fluoride-co-hexafluoropropylene) [20], nafion [21], and ammonium hydrogen difluoride [22]) to suppress the side reactions [23,24,25]. Another effective strategy is to construct an active site to guide Li^+^ plating and promote uniform Li deposition, such as using lithiophilic alloys [26] (Li-Sn [27], Li-Al [28], Li-In [29], Li-Zn [30]) to reduce heterogeneous nuclear barriers. However, fabricating stable and long-life Li anodes is remains a challenge. The main reason for this is that the above methods only focus on one kind of passivation layer or active site, which cannot guarantee the coordination of the passivation effect and active site effect simultaneously. Therefore, the continuous chemically stable interface and uniform distribution of Li^+^ in the whole electroplating process is critical to realize the long cycle life of LMBs.

Herein, we propose a dual functional artificial SEI with a simultaneous passivation and active site effect via introducing MgF_2_ on the polished surface of Li. We discover that a dual functional layer consisting of Li-Mg alloy and LiF are generated by in situ surface reactions between MgF_2_ with Li metal, and the two components synergically protect the Li metal anode from dendrite growth. The electronic-insulating LiF component prevents the electron conduction at the SEI/Li interphase to extend the cycling stability of LMBs. Meanwhile, the lithiophilic Li-Mg solid solution alloy serves as a nucleation active site to reduce Li^+^ diffusion barriers and forms ionic channels in the interphase layer, which guide the Li to deposit uniformly and suppress lithium dendrite prolongation. As a result, the electrochemical performance is greatly improved using the modified Li anode. The paired Li||Li symmetric cell exhibits an excellent plating/stripping stability for more than 2000 h with a flat polarization (12.0 mV) at 1.0 mA cm^−2^, which suggest the modified Li anode effectively restrains the stretch of dendrites during long cycling. Correspondingly, the effect of the modified Li anode on low interface impedance, high CE, and long lifespan of Li||Li and Li||LFP cells is investigated. This work provides a facial strategy for fabricating stable and longevous LMBs.

## 2. Results and Discussion

### 2.1. Construction of the Dual Functional Artificial Layer

To explore the effects of different concentrations of MgF_2_ in DME solvent on the cycling performance, the modified Li were treated with concentrations of 10 mM, 20 mM, and 50 mM (concentration-MF-Li). Additionally, the cycling stability of symmetric cells with different electrodes were carried out at 1.0 mA cm^−2^ with a capacity of 1.0 mAh cm^−2^. At an optimum soaking concentration of 50 mM, the Li||Li battery with the MF-Li anode presented the most stable cycling life of over 2000 h, as shown in Figure S1.

**Figure 1:**
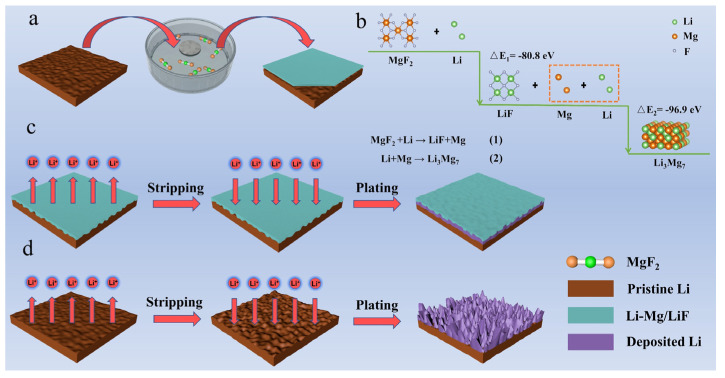
(**a**) Schematic for the fabrication procedure of MF-Li anode. (**b**) The enthalpy changes of chemical reactions testified by density functional theoretical (DFT) method. The morphology evolution illustration of the (**c**) MF-Li anode with MgF_2_ pre-treating process, and (**d**) pristine Li.

On the one hand, the Li-Mg solid solution alloy is identified as a lithiophilic site for Li^+^ plating with low nucleation overpotential to facilitate uniform deposition of Li^+^, and can serve as a beneficial stabilizer to stable the SEI layer [31]. On the other hand, LiF is generally considered as a favorable chemical module in the SEI layer to effectively passivate the Li/electrolyte interphase and contribute to the long-term cycling stability [32]. A dual functional artificial Li-Mg/LiF layer on the Li surface could be obtained through a replacement reaction between Li metal and MgF_2_-containning solution (Li + MgF_2_→LiF + Mg Li + Mg→Li_3_Mg_7_). The chemical reactions are favored due to the huge gap of enthalpy, calculated using the density functional theoretical (DFT) method. A schematic of the fabrication procedure for the MF-Li electrode is depicted in Figure 1.

**Figure 2:**
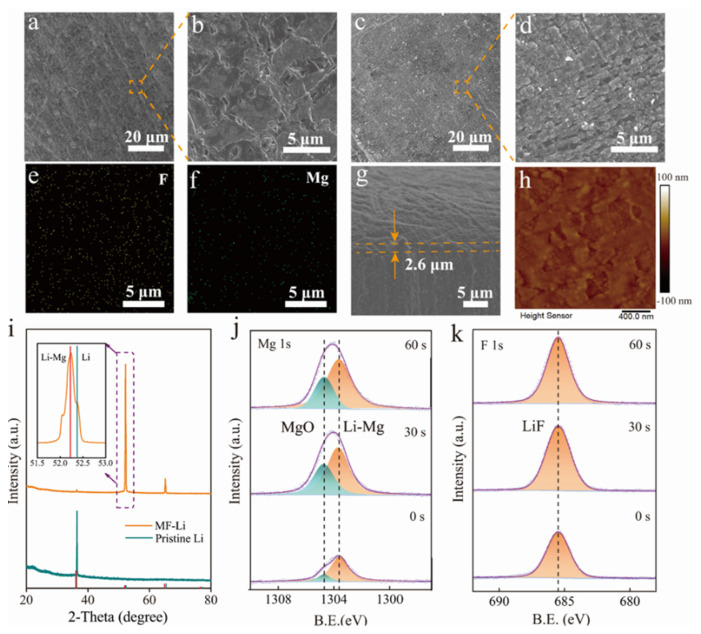
Characterization of the MF-Li anode. Top-view SEM images of the (**a**,**b**) pristine Li and (**c**,**d**) the MF-Li anode. EDS mapping images of (**e**) the F element and (**f**) the Mg element for the MF-Li anode. (**g**) The cross-sectional SEM image of the interphase. (**h**) AFM top view of the MF-Li anode. (**i**) X-ray diffraction pattern of the MF-Li and pristine Li anodes. High-resolution XPS depth profiles of (**j**) Mg 1s and (**k**) F 1s for the MF-Li anode.

Figure 2a–d displays the surface morphology images of pristine Li and the MF-Li anode. Obviously, pristine Li foil shows a mossy and rough surface. In comparison, the MF-Li anode shows a dense surface covered with block particles, which further proves that MgF_2_ reacts with Li metal to generate a dense protective layer. Energy dispersive spectroscopy (EDS) mapping of the interfacial layer reveals the homogeneous distribution of Mg and F elements (Figure 2e,f and Figure S2), favoring the establishment of the protective layer to promote uniform diffusion of Li^+^ through the SEI layer. From the SEM mapping spectra, the weight ratio of Mg is 39.16%, while the weight ratio of F is 60.84%. It is possible to calculate that the atomic ratio of Mg and F is 1:2. Therefore, the count of the F element is higher than that of Mg in the SEM mapping images. The cross-sectional view of the MF-Li anode shows that the thickness of artificial layer is 2.6 μm (Figure 2g). The atomic force microscopy (AFM) image also indicates that the surface of the modified layer is homogenous and dense, which is consistent with the SEM results of the MF-Li anode (Figure 2d,g). 

In addition, the compositions of the modified layer were analyzed by X-ray diffraction (XRD) characterization. The XRD pattern depicted in Figure 2i proves the existence of Li_3_Mg_7_ (Li-Mg) (PDF #65-6742) on the Li foil. Moreover, X-ray photoelectron spectroscopy (XPS) was performed to probe the surface chemical compositions of the MF-Li anode (Figure 2j,k). The peak at 685.5 eV in the F 1s region is ascribed to the presence of LiF. The Mg 1s spectrum region confirms the presence of Mg^0^ in the upper surface (1303.6 eV), indicating the existence of the metallic Li-Mg alloy phase of the MF-Li electrode. The peak at the binding energy of 1304.6 eV is attributed to MgO. This formation is related to the trace H_2_O in the DME solvent or oxygen in the surrounding air during the sample preparation process [33]. As the etching time increases, the content of Mg^0^ increases and MgO (1304.6 eV) decreases, which further implies the reaction between MgF_2_ and Li to generate LiF and the Li-Mg alloy. 

### 2.2. Mechanism of the Uniform Li^+^ Distribution

**Figure 3:**
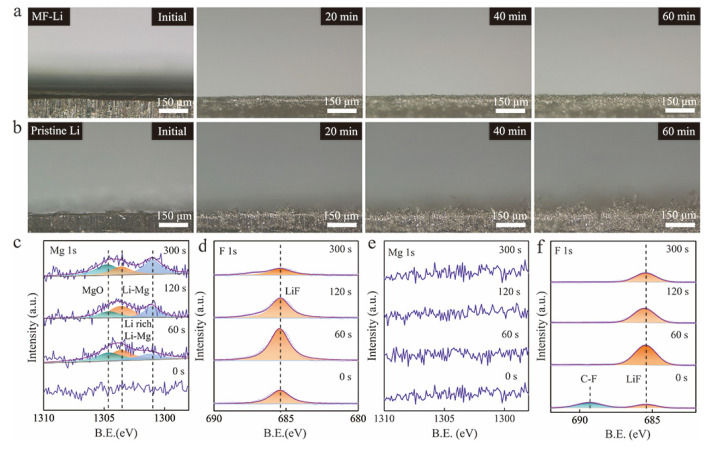
In situ optical microscopy visualization for the Li||Li cells with (**a**) the MF-Li anode and (**b**) the pristine Li anode. The scale bars are 150 μm. XPS depth analysis of the modified layer on MF-Li and pristine Li anodes after 5 cycles in the Li||Li cells: (**c**) Mg 1s and (**d**) F 1s spectra of the MF-Li anode; and (**e**) Mg 1s and (**f**) F 1s spectra of the pristine Li anode after different etching duration (depths).

In operando, optical microscopy was performed to intuitively visualize and record the morphological evolutions of the Li electrodeposition process at 3.0 mA cm^−2^ for 1 h. As depicted in Figure 3a, there is a compact and flat surface without dendrite during the whole Li deposition process of the MF-Li anode. In contrast, abundant Li needle-points appear after 20 min of plating. With the increase in deposition time, some needle-points grow into severe Li dendrites, resulting in loose and uneven interphase between the Li electrode and the electrolyte (Figure 3b). The dendrite-free Li deposition with the MF-Li anode demonstrates that the dual function artificial SEI layer can inhibit the lithium dendrites growth. 

In-depth XPS characterization was also performed to identify the SEI components of MF-Li and pristine Li anodes after five cycles of the Li plating/stripping process (Figure 3c–f). The characteristic peaks of the Li-Mg alloys and LiF are well presented, which provides additional evidence for the superior stability of the Li-Mg/LiF@Li anode. Figure 3c reveals that the Li-Mg alloy phase is not found before Ar^+^ etching. As the etching duration increases, major distinctive peaks in the Mg 1s spectrum are found at 1304.6, 1303.6, and 1301.0 eV. Compared to MF-Li before cycling (Figure 2j), in addition to the peaks of 1304.6 and 1303.6 eV attributed to MgO and the Li-Mg alloy, a new characteristic peak appears with lower binding energy, which corresponds to the Li-Mg solid solution alloy with higher Li content (Li-rich Li-Mg alloy). Furthermore, the content of Li-rich Li-Mg alloy increases with further etching, while the content of Li-Mg alloys gradually decreases. This phenomenon is attributed to the formed Li-rich Li-Mg alloy with increasing Li content, resulting from the further lithiation of the Li_3_Mg_7_ alloy in the Li plating/stripping process [34]. According to the reported literature [35], the Li-rich Li-Mg alloys can also guide the uniform Li deposition and effectively inhibit lithium dendrite growth [35,36,37]. As seen in Figure 3d, one main peak in the F 1s spectrum, at around 685.5 eV, is attributed to LiF. However, there is one more peak at about 689.3 eV existing in pristine Li metal, which is attributed to C-F bond. It is evident that the MF-Li electrode effectively inhibits the side reactions after pre-treatment. Consequently, the effects of the dual phase artificial SEI layer are confirmed by the above results. Both the Li-Mg alloy and LiF components play essential roles in improving the stability of the interphase during the Li plating and stripping process, which is consistent with the result of operando optical microscopy.

### 2.3. Electrochemical Evaluation of the Dual Functional Artificial Layer

Electrochemical impedance spectroscopy (EIS) analyses were implemented to investigate the stability of symmetric Li||Li batteries. As depicted in Figure S4, the impedance plots are composed of two parts of a depressed semicircle in the high and middle frequency area and a slope in the low frequency area. The resistance value of the high frequency zone at the intersection of the horizontal axis represents the body resistance R_b_, and the semicircle diameter represents interface resistance R_ct_, reflecting the ability of interfacial charge-transfer. In the beginning, the cell using the MF-Li anode presents a lower resistance (~59 Ω) than the pristine Li anode resistance (~94 Ω). The resistance of the cells increased to 70 Ω and 120 Ω for the MF-Li and the pristine Li anodes, respectively, after 5 days rest. Furthermore, after 20 days rest, the impedance of the battery with the MF-Li anode slightly increases to 78 Ω, while that of the pristine Li anode continuously increases to 160 Ω. The lower resistance of the MF-Li anode is attributed to the modified SEI layer with high ionic conductivity, which allows Li^+^ to diffuse quickly. The ionic conductivity of the artificial modified layer is based on the EIS test of MF-Li||MF-Li symmetrical cells at original state. The calculated ion conductivity of the Li-Mg/LiF layer is about 0.93 × 10^−5^ S cm^−1^. In contrast, the ion conductivity of defect-free LiF is around 10^−12^ S cm^−1^ [38], which is lower than that of artificial SEI. The results demonstrate that the interphase layer has a high ionic conductivity to allow Li^+^ ions to diffuse quickly and realize homogenous Li deposition on the anode.

To validate the advantages of the artificial interphase, the electrochemical performances of the modified Li anode were explored. Nucleation overpotential is obtained by calculating the potential difference between the lowest tip voltage and voltage polarization, and represents the energy barrier that Li^+^ deposition needs to overcome. Figure S5 displays that nucleation overpotential of MF-Li is 2.3 mV, much lower than that of bare Li (29.8 mV). The lower nucleation overpotential indicates that the LiF and Li-Mg alloy in the SEI allows Li^+^ to rapidly pass through the interphase and improves uniform Li nucleation. 

**Figure 4:**
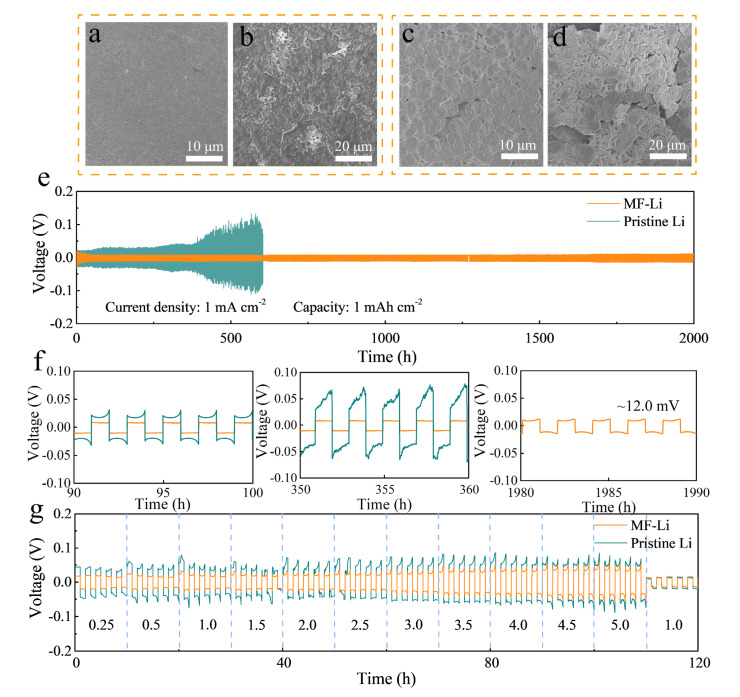
The surface morphology of (**a**) the MF-Li and (**b**) the pristine Li anode after plating at the current density of 0.5 mA cm^−2^ for 2 h. (**c**,**d**) Top view SEM images of (**c**) the MF-Li anode and (**d**) the pristine Li anode after 50 cycles in the symmetrical cells. (**e**) Voltage profiles of MF-Li and pristine Li anodes in the Li||Li symmetric cell at the current density of 1 mA cm^−2^ under a capacity of 1 mAh cm^−2^, and (**f**) the corresponding detailed voltage profiles. (**g**) Rate performances of MF-Li and pristine Li anodes measured at the current densities of 0.25, 0.5, 1, 1.5, 2, 2.5, 3, 3.5, 4, 4.5, and 5 mA cm^−2^ with each Li plating/ stripping process for 1.0 h.

Since it has been demonstrated that the Li-Mg solid solution alloy and LiF layer can efficiently inhibit the development of Li dendrite owing to the increased ions diffusivity and improved stability of the interphase, the surface morphology evolution of MF-Li and pristine Li were represented after plating at 0.5 mA cm^−2^ for 2 h. Figure 4a shows that a smooth and compact Li deposition layer is attached to the MF-Li metal, while the blank Li anode shows an uneven surface of mossy Li (Figure 4b). These morphology views indicate that the Li-Mg/LiF artificial SEI layer is conducive to homogeneous Li^+^ flux and dense morphologies of lithium deposition. In addition, the interface stability has significant effects on extending the cycling lifespan of LMBs. Moreover, the morphology and composition of the interphase changes constantly during the process of the battery running. The morphology evolution of the Li anode with and without treatment in Li||Li cells after 50 cycles is presented in Figure 4c,d and Figure S6. The existing Li-Mg alloy and LiF layer forms an even and dense Li deposition layer, while abundant dendrites emerge on the primitive Li anode.

Furthermore, the cycling stability of the modified electrode was evaluated by symmetric MF-Li||MF-Li cells at 1 mA cm^−2^ with a capacity of 1 mAh cm^−2^. The voltage-time profiles depicted in Figure 4e reveal that the MF-Li||MF-Li cells maintain stable for more than 2000 h, which is significantly preferable to the pristine Li||Li cells, whose overpotential gradually increases after 300 h. The excellent cycling stability is also superior to some reported works, as shown in Table S1. In addition, as depicted in Figure 4f, the stable overpotential of the MF-Li||MF-Li cell (~12.0 mV) is lower than that of the pristine-Li||Li cell (~30.3 mV). When increasing the current density to 5 mA cm^−2^, the cells paired with the MF-Li electrode maintain stable cycling for over 250 cycles, which is superior to the unstable voltage polarization variation of the pristine Li anode (Figure S8). The low Li plating/striping plateau of the MF-Li||MF-Li cell indicates that the Li^+^ can quickly diffuse through the interphase and superior stability of the SEI layer. Furthermore, the artificial SEI layer conduces to enhance the electrochemical stability of symmetric batteries at different current densities. As depicted in Figure 4g, the battery with the MF-Li electrode exhibits superior stability and lower voltage polarization (from 14.5 to 18.1, 23.9, 27.3, 38.2, 45.3, to 52.3 mV) at the current density ranging from 0.25 to 5.0 mA cm^−2^. These satisfied results are attributed to effective regulation of Li^+^ flux on the artificial interphase by LiF and the Li-Mg alloy.

### 2.4. Battery Performance

**Figure 5:**
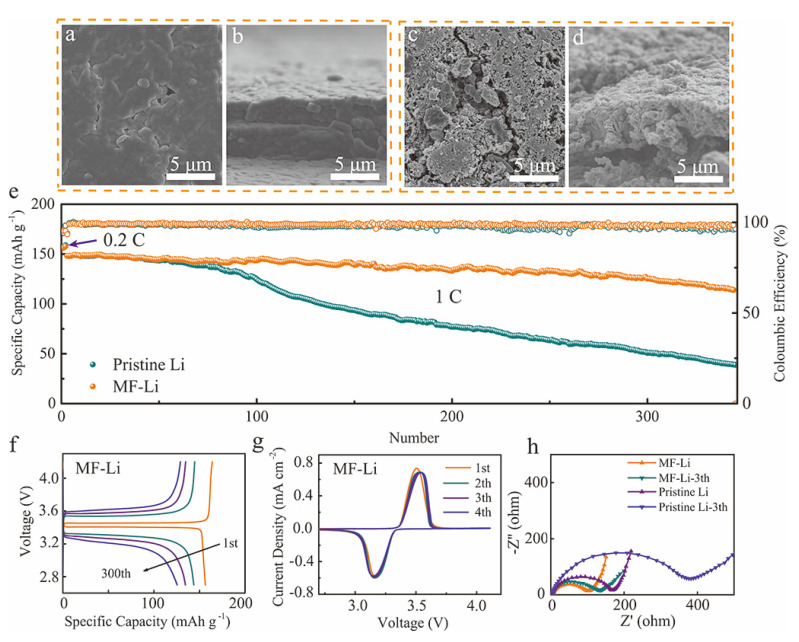
Cycling performance of the MF-Li||LFP cell and the Li||LFP cell at the rate of 1 C. Top view and the cross-section SEM images of (**a**,**b**) the MF-Li anode and (**c**,**d**) the pristine Li anode after 30 cycles. (**e**) Cycling stabilities of full cells with the MF-Li and pristine Li anodes. (**f**) The charge/discharge profiles at different cycles of the MF-Li||LFP cell. (**g**) CV curves of the MF-Li||LFP cell at the scan rate 0.1 mV s^−1^. (**h**) EIS spectra of the Li||LFP cells with different anodes at the original state and after three cycles.

To further investigate the practicability of the MF-Li and pristine Li anodes, LiFePO_4_ (LFP) was used as cathode and the cycling stability of the full batteries were evaluated at 1 C (1 C = 170 mAh g^−1^). Figure 5a–d shows the surface and cross-section views of the two kinds of anodes after 30 cycles in a discharge process. The MF-Li anode has a smooth surface, indicating no severe interfacial variations have taken place. The cross-sectional morphology view of the Li deposition with the artificial SEI layer is dense and even, suggesting that the Li-Mg solid solution alloy guides the Li deposition and reduces volume fluctuations. As cycling proceeds, both cells exhibit stable coulombic efficiency (CEs). However, the MF-Li||LFP battery displays longer cycling life than the Li||LFP battery (Figure 5e). After 300 cycles, the battery with the MF-Li anode still maintains a satisfied specific capacity of ~125.2 mAh g^−1^, achieving capacity retention of about 84.6%, while the capacity of Li||LFP battery continuously fades after 100 cycles, and drops to only 50.9 mAh g^−1^. The corresponding charge-discharge profiles are presented in Figure 5f and Figure S9. The MF-Li||LFP battery retains consistent voltage polarization and high specific capacity in the cycling process, indicating that the dual functional layer efficiently regulates Li deposition and suppresses dendrite growth. In comparison, the pristine-Li||LFP battery reveals increased voltage polarization and much lower capacity retention owing to the unstable SEI layer. Figure S11 shows the cycling performance of Li||LFP batteries with increased loading of 5.4 mg cm^−2^ under a rate of 1 C. The Li||LFP battery assembled with the MF-Li anode still shows a higher specific capacity and CEs than the cell with the pristine Li anode during cycling, which is attributed to the rapid Li^+^ transport in the dual functional artificial SEI layer, while the Li||LFP battery with the pristine Li anode displays a continuously decreasing CE, caused by lithium dendrites and “dead” Li on the Li anode surface. The rate performance was conducted at different current densities from 0.2 C to 5 C, as shown in Figure S10. Based on the dual functional artificial SEI layer, the full cell with the MF-Li anode delivers excellent rate capability. At different rates, all batteries have a higher specific capacity (160, 154.5, and 129.7 mAh g^−1^ at 0.2, 1, 5 C, respectively) than those with the pristine Li anode (154.1, 144.6, and 107.4 mAh g^−1^ at 0.2, 1, 5 C, respectively).

The cyclic voltammetry (CV) profiles of Li||LFP batteries paired with different anodes are compared in Figure 5g and Figure S12. The almost overlapped CV profiles of the MF-Li||LFP cell indicates the highly reversible electrochemical reaction and prominent cycling stability. Inversely, the CV profiles of the pristine-Li||LFP cell show that the voltage discrepancy between the oxidation peak and the reduction peak gradually enlarges during cycling, indicating an increased polarization. Thus, this leads to the poor reversibility and cycle performance. This result matches with the cell performance of the Li||LFP batteries (Figure 5e). The EIS plots of the MF-Li||LFP and the Li||LFP cells were also measured at the origin state and after 3 cycles, as seen in Figure 5h. The result indicates that the anode with the modified layer exhibits a lower resistance. In the beginning, the cell using the MF-Li anode presents a lower resistance of about 96.8 Ω compared to the pristine Li anode resistance (163.3 Ω). This is attributed to the protective effect of the Li-Mg/LiF artificial layer, which prevents the side reaction between the Li anode and the electrolyte. However, the high activity of the pristine Li anode can react with the ester electrolyte to form an unstable and fragile SEI layer, which increases the resistance of the battery. After three cycles, the impedance of the battery with the modified layer slightly increases to 129.2 Ω due to the good stability of the Li-Mg/LiF modified layer. In comparison, the impedance value after three cycles increases significantly for the cell without a modified layer, which is caused by instability of SEI generated by the harsh side reaction. The EIS spectra further suggest the effective cooperating role of the Li-Mg alloy and LiF in constructing a stable artificial SEI layer during cycling.

## 3. Experimental Section

### 3.1. Materials

Magnesium fluoride (MgF_2_, 99.8%) was obtained from Sigma-Aldrich (Shanghai, China). Dimethyl ether (DME) was purchased from Alfa Aesar (Shanghai, China). The ether electrolyte consisting of 1.0 M bis(trifluoromethane) sulfonimide lithium (LiTFSI) with 1.0% LiNO_3_ in 1,3-dioxolane (DOL) and dimethoxyethane (DME) solvents (*v*/*v*, 1:1) was configurated from DODOChem (Suzhou, China). In addition, the ester electrolyte consisting of 1.0 M lithium hexafluorophosphate (LiPF_6_) and ethylene carbonate (EC)/diethyl carbonate (DEC)/ethyl methyl carbonate (EMC) (*v*:*v*:*v*, 1:1:1) was configurated from DODOChem. All reagents were used directly without purification.

### 3.2. Electrode Preparation

The pristine Li foil (99.9%, 0.5 mm) was firstly polished to remove surface contaminants by rolling. Then, 31.2 mg MgF_2_ of powder were dissolved in 10 mL DME solvent under vigorous stirring. After being dipped in the MgF_2_ solution for 30 s and resting overnight, the color of the Li foil surface turns gray. Then, the residual MgF_2_ on the Li metal surface was cleaned with DME, and dried overnight under Ar atmosphere. Finally, the Li-Mg/LiF biphasic layer on the Li metal was obtained.

Next, 240 mg lithium iron phosphate (LiFePO_4_, LFP) particles, 30 mg Super-P, and 30 mg PVDF were dispersed in *N*-methyl-2-pyrrolidinone (NMP) solvent and ball milled for 30 min, forming a uniform suspending liquid. Then, the suspending liquid was cast on the Al current collector and then dried under 60 °C for one night. The prepared electrode was cut into pieces (11.0 mm) for future use. The mass loading of cathode materials was about 2.4 mg cm^−2^. More detailed content of materials characterizations, electrochemical measurements, and the computational method are supplied in the supporting information.

## 4. Conclusions

In conclusion, through the construction of the Li-Mg alloy and the LiF dual functional artificial protective layer by facile surface chemistry, we successfully decreased the interfacial resistance of the Li anode and reduced the Li deposition overpotential, extending the cycling lifetime of LMBs. The LiF component with the passivation effect can effectively hinder the parasitic reactions between the Li anode and the electrolyte to promote cycling stability, while the lithophilic Li-Mg solid solution alloys that exhibit the active site effect can reduce the Li nucleation barrier and homogenize the Li^+^ deposition. The excellent electrochemical stability of the interphase characterized by SEM images suggests that the modified layer can effectively prevent the growth of lithium dendrite during repeated cycling. Thus, the outstanding cycling performance of the MF-Li||MF-Li symmetric cell is achieved: a long-term cycling stability (of over 2000 h) with low voltage hysteresis (12.0 mV) at a current density of 1 mA cm^−2^. The MF-Li||LFP battery also shows superior capacity retention of 84.6% for over 300 cycles at a rate of 1 C. These cycling performances reveal the beneficial synergistic effect of the Li-Mg alloy and LiF, enabling homogeneous deposition of Li metal. In light of the aforementioned benefits, the dual functional artificial SEI layer is considered as a potential candidate for practical applications of high-energy rechargeable LMBs.

## Data Availability

Not applicable.

## Supplementary Materials

The following supporting information can be downloaded at: https://www.mdpi.com/article/10.3390/molecules27165199/s1, Figure S1: Voltage profiles of MF-Li anodes with different pre-treating concentrations in the Li||Li symmetric cell at a current density of 1 mA cm^−2^ under a capacity of 1 mAh cm^−2^; Figure S2: EDS mapping images of Mg and F elements for the cross-sectional of the MF-Li anode; Figure S3: AFM top view of the pristine Li anode; Figure S4: Static impedance of Li||Li cells with different anodes at the original state, 12 h, 24 h, and 5 and 20 days rest; Figure S5: Nucleation overpotentials of the MF-Li and pristine Li anodes at the current density of 1 mA cm^−2^ with a capacity of 1 mAh cm^−2^; Figure S6: The surface morphology of (a) the MF-Li anode and (b) the pristine Li anode after plating at the current density of 0.5 mA cm^−2^ for 2 h; Figure S7: Cross-sectional view SEM images of (a) the MF-Li anode and (b) the pristine Li anode after 50 cycles in the symmetrical cells; Figure S8: Voltage profiles of MF-Li and pristine Li anodes in the Li||Li symmetric cell at the current density of 5 mA cm^−2^ under a capacity of 1 mAh cm^−2^ (insets are detailed voltage profiles); Figure S9: The voltage profiles of the Li||LFP cell with the pristine Li anode at different cycles; Figure S10: Rate performance of Li||LFP paired with the MF-Li and pristine Li anodes; Figure S11: Cycling stabilities of full cells with the two anodes under the conditions of 5.4 mg cm^−2^ and 1 C; Figure S12: CV curves of Li||LFP cell with the pristine Li anode at a scan rate 0.1 mV s^−1^; Table S1: Comparison of cycling stability of our work with the reported artificial SEI in symmetric cells and Li||LFP full cells. References [39,40,41,42,43,44,45,46,47,48,49,50] are cited in the supplementary materials.
