# Supplementary material for: A Dual Functional Artificial SEI Layer Based on a Facile Surface Chemistry for Stable Lithium Metal Anode
Source: Molecules. 2022 Aug 15;27(16):5199. doi: 10.3390/molecules27165199 (PMC9412686; doi:10.3390/molecules27165199)
Supplement: Supplementary file 1 [file molecules-27-05199-s001.zip › molecules-1837637-supplementary.pdf]

A Dual Functional Artificial SEI Layer Based on a Facile Surface Chemistry for Stable Lithium Metal Anode

*Yue Ma [a,c], Feng Wu [a,b,c], Nan Chen * [a,c], Tianyu Yang [a], Yaohui Liang [a,c], Zhaoyang Sun [a], Guangqiu Luo [d], Jianguo Du [d], Yanxin Shang [a,c], Mai Feng [a], Ziyue Wen [a,c], Li Li [a,b,c], and Renjie Chen* [a,b,c]*

[a]Beijing Key Laboratory of Environmental Science and Engineering, School of Materials Science and Engineering, Beijing Institute of Technology, Beijing 100081, China

[b]Collaborative Innovation Center of Electric Vehicles in Beijing, Beijing 100081, China

[c]Advanced Technology Research Institute, Beijing Institute of Technology, Jinan 250300, China

[d]The 18th Research Institute of China Electronics Technology Group Corporation, Tianjin300384, China

1. Experimental section

1.1. Materials Characterizations

The chemical composition of the artificial SEI layer was examined by an Rigaku Ultima IV-185 X-ray diffractometer (XRD) with Cu-Kα radiation. The morphology and structure of modified Li, pristine Li, and Li deposition were observed using a HITACHI S-3500N scanning electron microscope (SEM). Elemental species and chemical valence of samples surface were investigated by a PHI QUANTERA-II SXM X-ray photoelectron spectroscopy (XPS) with a monochromatic Al Kα X-ray source (1486.6 eV) for excitation. The XPS depth profiles are obtained by the bombardment of Ar-ion sputtering to study the in-depth composition distributions. The surface morphology of modified Li and blank Li were obtained by a Buker Icon atomic force microscope (AFM). Operando optical microscopy observations were recorded in a customized sealed electrochemical cell with transparent quartz window by an optical microscopy (Leica DVM6).

1.2. Electrochemical measurements

To explore the morphology and structure of Li deposition and Li plating/stripping cycling stability of different electrodes, CR2016-coin cells were assembled with MF-Li and pristine Li as working electrodes in an Ar-filled glove box. The thickness of the lithium anode used is 0.5 mm. The separator was Celgard 2325 with thickness of 25 μm. The electrolyte was 1.0 M bis(trifluoromethane) sulfonimide lithium (LiTFSI) with 1.0% $LiNO_3$ in 1,3-dioxolane (DOL) and dimethoxyethane (DME) solvents (v/v, 1:1). The amount of electrolyte was used 60 μL in each cell.

To evaluate the function of artificial SEI layer for the full cells, CR2025-coin cells were assembled with LFP cathode and Li anode. The separator and lithium anode were same as those used in symmetric batteries. The electrolyte was 1.0 M lithium hexafluorophosphate ($LiPF_6$) and ethylene carbonate (EC)/

diethyl carbonate (DEC)/ ethyl methyl carbonate (EMC) (v:v:v, 1:1:1). The amount of electrolyte was used 60 μL in each cell. All the cells were assembled in an Ar-filled glove box with the O_2 and H_2O content below 0.01 ppm. Charge-discharge cycling was tested on a Neware cell tester (CT-4008T, Shenzhen Neware Technology Company). Electrochemical impedance spectroscopy (EIS) measurements were performed on an electrochemical workstation with the frequency range of 100 kHz to 10 mHz (CHI660E, Shanghai Chenhua Company). To investigate the ionic conductivity of artificial SEI layer, the EIS measurement of Li||Li cells were performed, and the relative equation to calculate ionic conductivity of the modified SEI as follows [31]:

$$\sigma = 2L/(R \cdot S)$$

where L is the thickness of artificial layer, R is the interface resistance, and S is the contact area of modified Li anode.

1.3. Computational method

All the density functional theory (DFT) calculations were performed in generalized gradient approximation (GGA) using the PBE [34] by Vienna AB-Initio Simulation Package (VASP) [32,33]. The projected augmented wave (PAW) potentials [35,36] were chosen to depict the ionic cores. The plane wave basis with a kinetic energy cutoff of 400 eV was used to consider the valence electrons. The energy variation less than 10^{-5} eV meant that the electron energy was self-consistent. The force variation smaller than 0.01 eV/Å represented that geometry optimization was convergent. The enthalpy difference is calculated by subtracting the enthalpy of the reactants from the products.

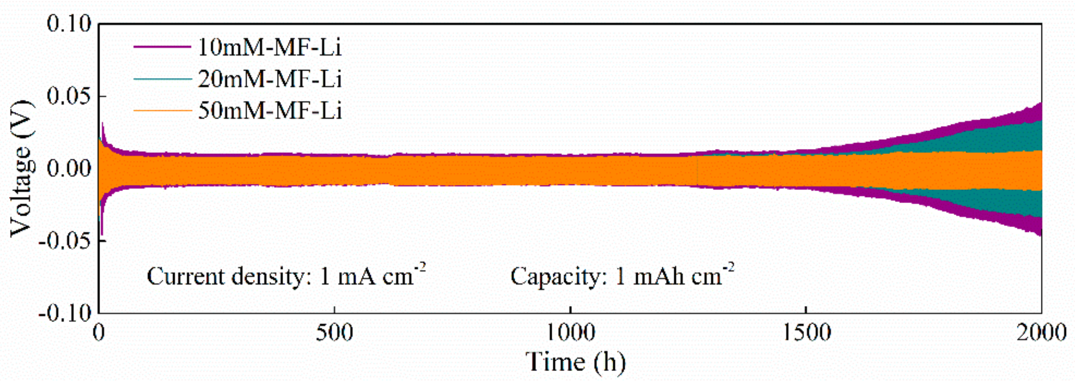

Figure S1. Voltage profiles of MF-Li anodes with different pre-treating concentration in Li||Li symmetric cell at the current density of 1 mA cm^{-2} under a capacity of 1 mAh cm^{-2}

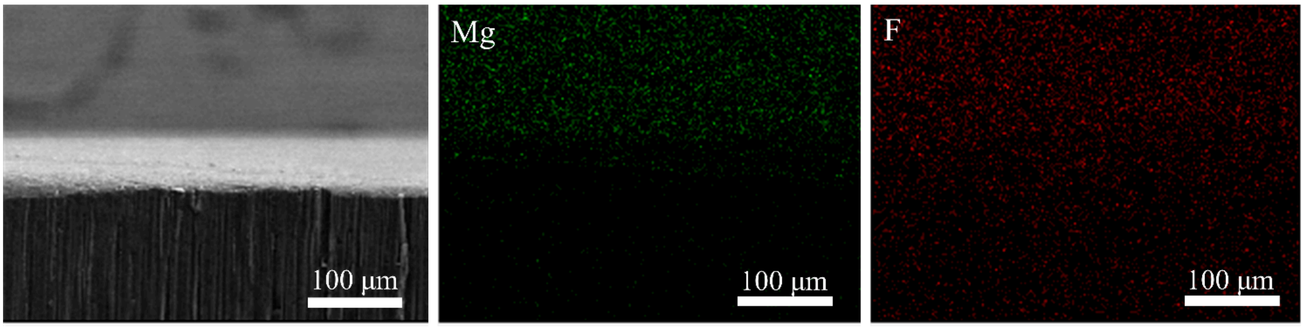

Figure S2. EDS mapping images of Mg and F elements for the cross-sectional of MF-Li anode.

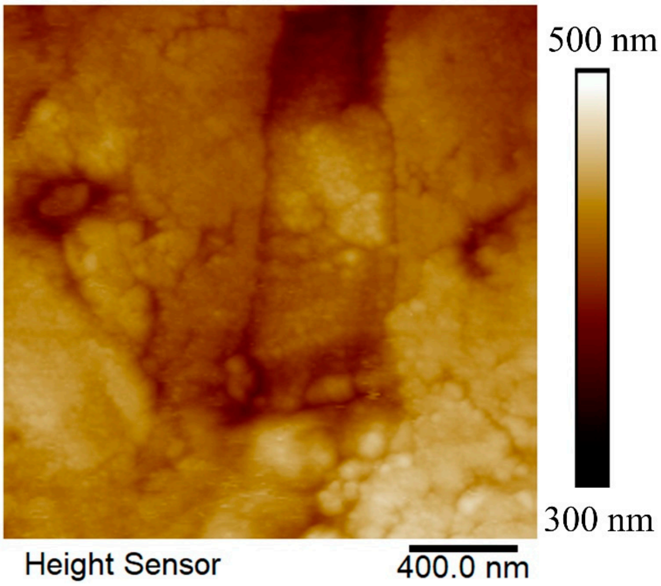

Figure S3. AFM top view of pristine Li anode.

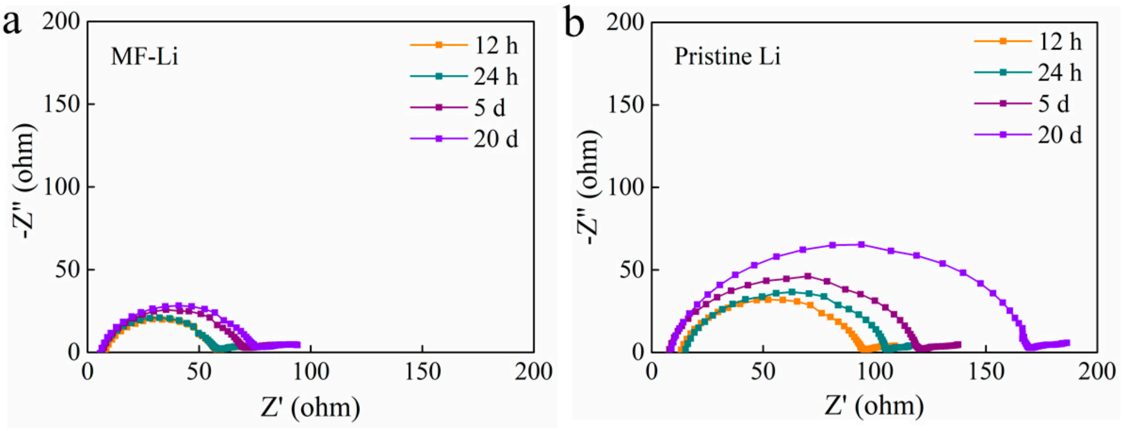

Figure S4. Static impedance of Li||Li cells with different anodes at original state, 12h, 24h, 5 and 20 days rest.

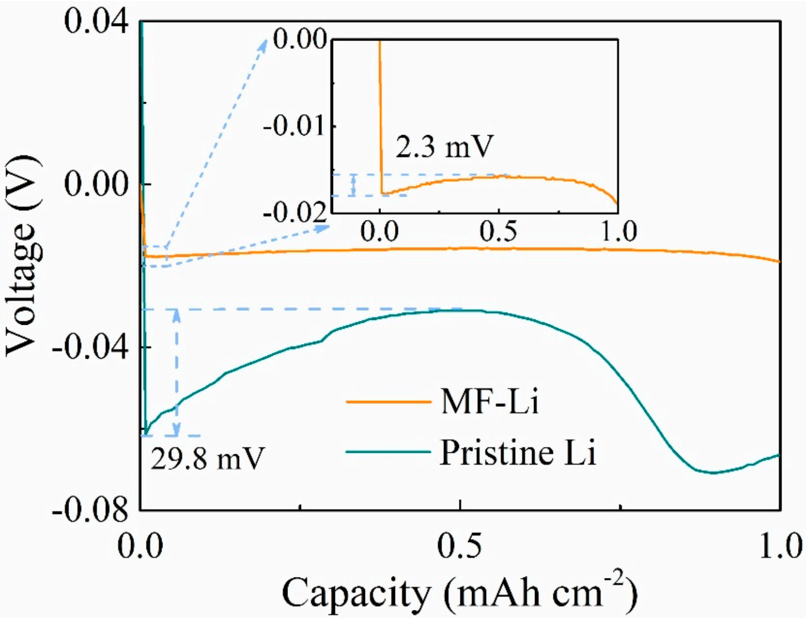

Figure S5. Nucleation overpotentials of MF-Li and pristine Li anode at the current density of 1 mA cm^{-2} with a capacity of 1 mAh cm^{-2}.

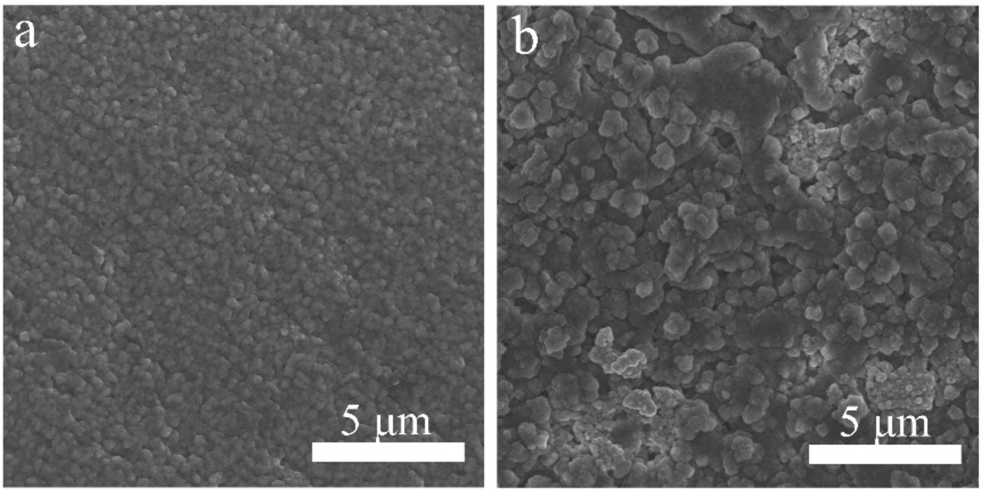

Figure S6. The surface morphology of MF-Li (a) and pristine Li anode (b) after plating at the current density of 0.5 mA cm^{-2} for 2 h.

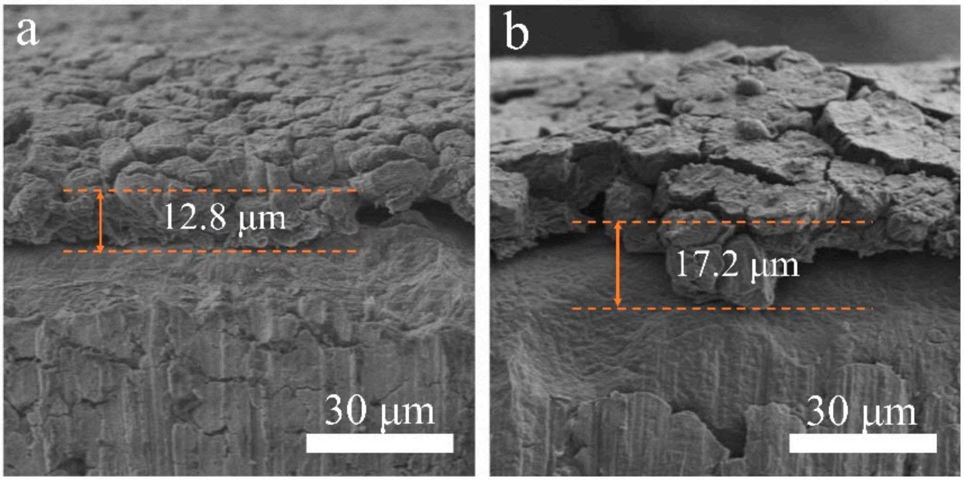

Figure S7. Cross-sectional view SEM images of (a) MF-Li anode and (b) pristine Li anode after 50 cycles in the symmetrical cells.

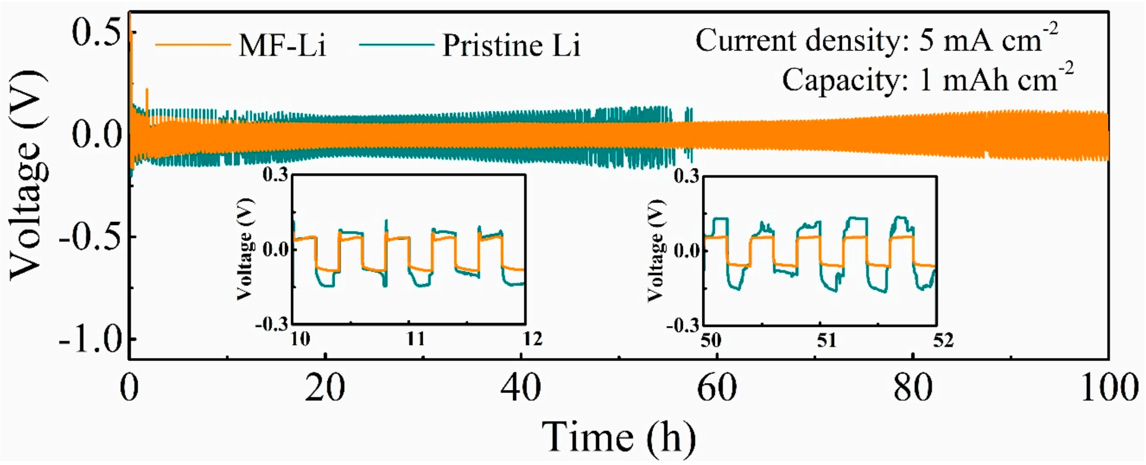

Figure S8. Voltage profiles of MF-Li and pristine Li anodes in Li||Li symmetric cell at the current density of 5 mA cm^{-2} under a capacity of 1 mAh cm^{-2} (insets are detailed voltage profiles)

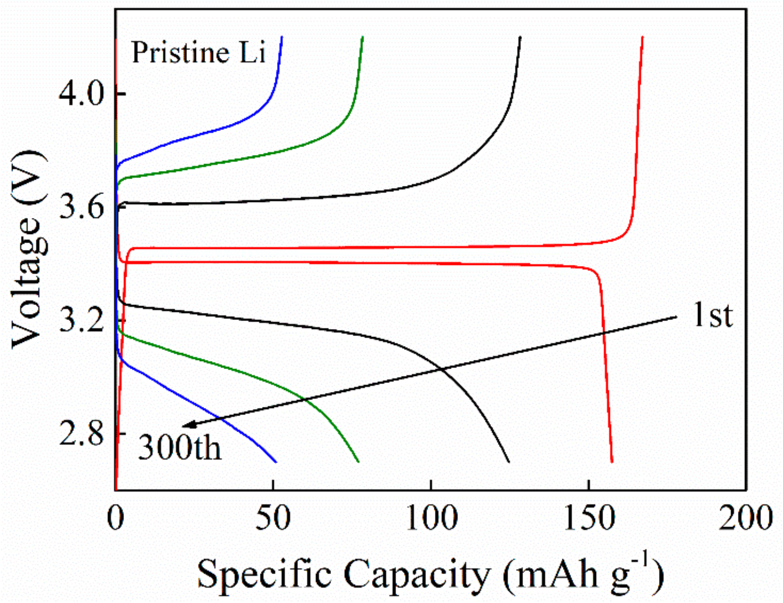

Figure S9. The voltage profiles of Li||LFP cell with pristine Li anode at different cycles.

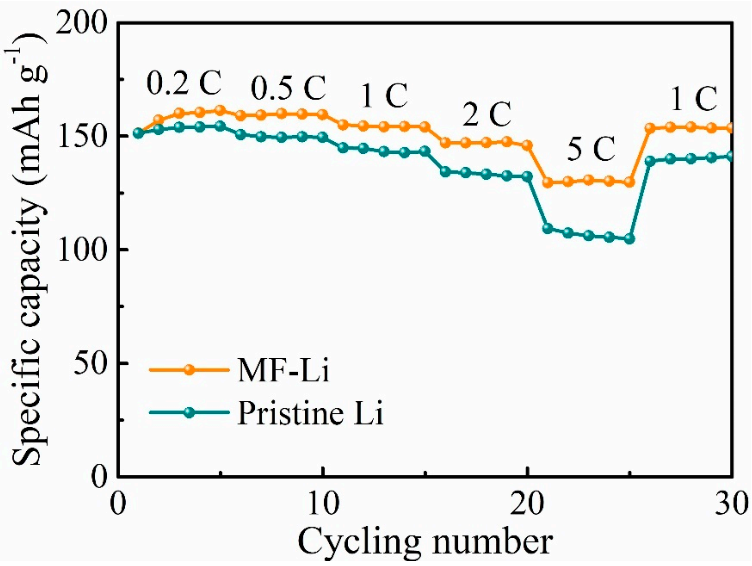

Figure S10. Rate performance of Li||LFP paired with MF-Li and pristine Li anodes.

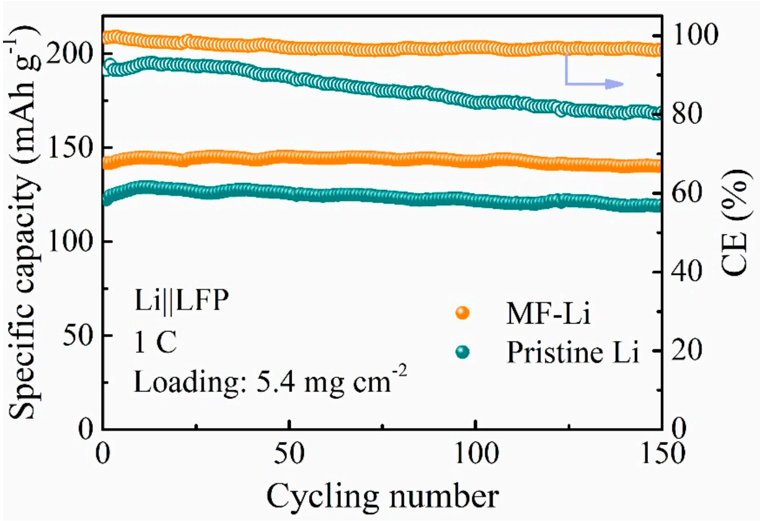

Figure S11. Cycling stabilities of full cells at 1 C with MF-Li and pristine Li as anode paired with cathode loading of 5.4 mg cm^{-2}.

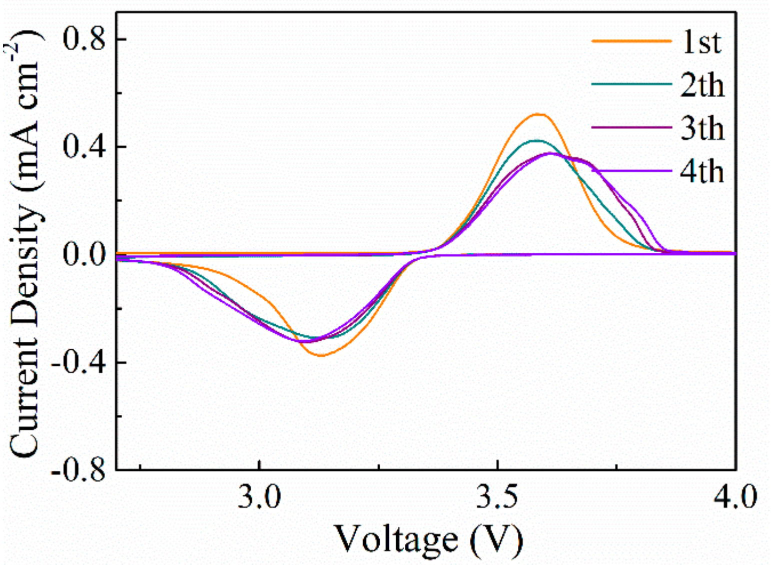

Figure S12. CV curves of Li||LFP cell with pristine Li anode at a scan rate 0.1 mV s^{-1}.

Table S1. Comparison of cycling stability of our work with the reported artificial SEI in symmetric cells and Li||LFP full cells.

**Modified SEI**	**Symmetric Li||Li cells ($mA\ cm^{-2}$/$mAh\ cm^{-2}$)**	**Li||LFP cells (rate/cycles/capacity)**	**Ref**
LiZn@Li	900 h (1/1)	0.5 C, 200 cycles, 130 mAh g^{-1}	[7]
LiSb/LiF@Li	700 h (1/1)	Not mentioaned	[8]
LiZn/Li_3PO_4@Li	600 h(1/1)	1 C, 300 cycles, 85 mAh g^{-1}(64%)	[9]
PVDF-HFP/LiF@Li	600 h(1/1)	Not mentioaned	[10]
LiSe@Li	1000 h(1/1)	Not mentioaned	[11]
Li_3PS_4@Li	800 h(1/1)	1 C, 145 cycles, 64%	[12]
GF/LiF@Li	800 h(1/1)	1 C, 200 cycles, 135 mAh g^{-1}	[13]
LiMg/LiF@Li	2000 h(1/1)	1 C, 200 cycles, 135.6 mAh g^{-1}(91.8%)	**This work**

[1] Hu, A.; Chen, W.; Du, X.; Hu, Y.; Lei, T.; Wang, H.; Xue, L.; Li, Y.; Sun, H.; Yan, Y.; Long, J.; Shu, C.; Zhu, J.; Li, B.; Wang, X.; Xiong, J. An artificial hybrid interphase for an ultrahigh-rate and practical lithium metal anode. *Energ Environ Sci.* **2021**, *14*, 4115-4124.

[2] Perdew, J. P.; Burke, K.; Ernzerhof, M. Generalized gradient approximation made simple. *Phys. Rev. Lett.* **1996**, *77*, 3865.

[3] Kresse, G.; Furthmüller, J. Efficiency of ab-initio total energy calculations for metals and semiconductors using a plane-wave basis set. *Comput. Mater. Sci*. **1996**, *6*, 15.

[4] Kresse, G.; Furthmüller, J. Efficient iterative schemes for ab initio total-energy calculations using a plane-wave basis set. *Phys. Rev. B.* **1996**, *54*, 11169.

[5] Kresse, G.; Joubert, D. From ultrasoft pseudopotentials to the projector augmented-wave method. *Phys. Rev. B.* **1999**, *59*, 1758.

[6] Blochl, P. E. Projected augmented-wave method. *Phys. Rev. B.* **1994**, *50*, 17953-17979.

[7] Chen, Q.; Li, H.; Meyerson, M. L.; Rodriguez, R.; Kawashima, K.; Weeks, J. A.; Sun, H.; Xie, Q.; Lin, J.; Henkelman, G.; Heller, A.; Peng, D. L.; Mullins, C.B. Li-Zn overlayer to facilitate uniform lithium deposition for lithium metal batteries. *ACS Appl Mater Interfaces.* **2021**, *13*(8), 9985-9993.

[8] Cui, C.; Zhang, R.; Fu, C.; Xiao, R.; Li, R.; Ma, Y.; Wang, J.; Gao, Y.; Yin, G.; Zuo, P. Stable lithium anode enabled by biphasic hybrid SEI layer toward high-performance lithium metal batteries. *Chem Eng J*. **2022**, *433*, 133570.

[9] Wang, X.; Zhuang, J.; Liu, M.; Wang, C.; Zhong, Y.; Wang, H.; Cheng, X.; Liu, S.; Cao, G. Facile and scalable engineering of a heterogeneous microstructure for uniform, stable and fast lithium plating/stripping. *J. Mater. Chem. A.* **2019**, *7*(32), 19104-19111.

[10] Li, S.; Fan, L.; Lu, Y. Rational design of robust-flexible protective layer for safe lithium metal

battery. *Energy Stor. Mater.* **2019**, *18*, 205-212.

[11] Ma, Y.; Wei, L.; Gu, Y.; Zhao, L.; Jing, Y.; Mu, Q.; Su, Y.; Yuan, X.; Peng, Y.; Deng, Z. Insulative ion-conducting lithium selenide as the artificial solid-electrolyte interface enabling heavy-duty lithium metal operations. *Nano Lett*. **2021**, *21*(17), 7354-7362.

[12] Wang, H.; Wu, L.; Xue, B.; Wang, F.; Luo, Z.; Zhang, X.; Calvez, L.; Fan, P.; Fan, B. Interfaces, improving cycling stability of the lithium anode by a spin-coated high-purity Li_3PS_4 artificial SEI layer, *ACS Appl. Mater. Interfaces.* **2022**, *14*(13), 15214-15224.

[13] Shen, X.; Li, Y.; Qian, T.; Liu, J.; Zhou, J.; Yan, C.; Goodenough, J. Lithium anode stable in air for low-cost fabrication of a dendrite-free lithium battery. *Nat. Commun.* **2019**, *10*(1), 1-9.
